# Effect of *Passiflora Edulis* Sims Peel Feed on Meat Quality of Finishing Pigs

**DOI:** 10.3390/foods14040561

**Published:** 2025-02-08

**Authors:** Xueying Zai, Xianyong Ma, Guangying Weng, Min Song, Yusheng Lu, Liyi Yang, Dun Deng

**Affiliations:** 1 College of Animal Science & Technology, Zhongkai University of Agriculture and Engineering, Guangzhou 510225, China; zxying214@163.com; 2 State Key Laboratory of Livestock and Poultry Breeding, Key Laboratory of Animal Nutrition and Feed Science in South China, Ministry of Agriculture and Rural Affairs, Guangdong Provincial Key Laboratory of Animal Breeding and Nutrition, Guangdong Engineering Technology Research Center of Animal Meat quality and Safety Control and Evaluation, Institute of Animal Science, Guangdong Academy of Agricultural Sciences, Guangzhou 510640, China; maxianyong@gdaas.cn (X.M.); wengguangying123@163.com (G.W.); songmin@gdaas.cn (M.S.); 3 Guangdong Key Laboratory of Nutrient Cycling and Farmland Conservation, Institute of Agricultural Resources and Environment, Guangdong Academy of Agricultural Sciences, Guangzhou 510640, China; luyusheng1@gdaas.cn; 4 Guangzhou Daqiao Food Equipment Co., Ltd., Guangzhou 510640, China; gzdaqiao@163.com

**Keywords:** *Passiflora edulis Sims* peel, meat quality, carcass traits, proteomic analysis, finishing pigs

## Abstract

*Passiflora edulis Sims peel* (Chinese name Baixiangguo, BXG) is a by-product with a high nutritional and economic value of Passiflora edulis Sims. In this study, corn was partly replaced with BXG to make feed for finishing pigs and the effects on the carcass traits, meat quality, muscle amino acid profile, and gene expression of finishing pigs were evaluated. A total of 20 healthy finishing pigs (Duroc × Landrace × Large) were randomly divided into two groups. The control group (CON) was fed the basal diet, and the experimental group (BXG) was fed a basal diet with BXG instead of 10% corn for a period of 43 d. Compared to the CON group, the carcass weight, intramuscular fat content, and marbling score were significantly increased, while the drip loss, b^*^ value, and shear force of the BXG group were significantly reduced (*p* < 0.05). Gene expression analysis showed that the mRNA expression of lipid synthesis and oxidative-type fiber related genes was significantly increased in the BXG group (*p* < 0.05). Proteomic research revealed that the metabolic pathways of the BXG and CON groups differed significantly. A total of 36 differentially expressed proteins were identified, mainly related to energy metabolism, fatty acid degradation, and endocrine regulation pathways. However, the contents of glutamine, glutamate, proline, and other amino acids in the BXG group were significantly reduced (*p* < 0.05). Overall, this study has a positive effect on improving meat quality, but the specific mechanism needs to be further explored, which offers practical guidance for the application of BXG in producing higher-quality pork and further promotes its commercial application.

## 1. Introduction

The market is seeing an increase in demand for high-quality pork as people’s living levels rise, presenting challenges in both market and production dimensions. These challenges include shifts in consumer preferences, escalating costs, heightened market competition, and difficulties in breeding and disease control [1]. Large-scale farming has the benefit of being able to lower production costs and boost production efficiency. However, the quality of pork is declining, evidenced by a reduction in pH levels and beneficial fatty acid content. Additionally, there may be potential impacts on meat quality attributes through the regulation of related gene expression [2,3,4]. Therefore, addressing these challenges through nutritional means has become the key to improving meat quality without altering the breed or feeding technique, such as adding probiotics, trace elements, and plant active substances [5].

Utilizing plant waste and agricultural processing by-products as animal feed additives has become an important research direction for enhancing meat quality. These natural resources not only help reduce environmental pollution but also enhance the sensory of meat through their rich nutritional content and bioactive substances. For instance, studies found that the content of antioxidant substances in pork can be enhanced, the lipid oxidation can be delayed, and the meat color and flavor can be improved by adding the proper tea residue to pig feed [6]. The antioxidant mechanisms in tissues can be increased, and lipid and protein damage induced by oxidative stress can be reduced by adding grape marc to weaned piglet feed, thereby improving the meat quality of piglets [7]. The quality of meat was greatly enhanced by using plant waste and agricultural industry by-products as animal feed additives. Nevertheless, a lot of nutritious agricultural processing by-products are thrown away, causing resource waste.

*Passiflora edulis Sims* peel (Chinese name Baixiangguo, BXG) constitutes approximately 50–60% of the fruit’s total weight. During juice extraction, these peels are typically discarded, resulting in a significant waste of resources. Studies have shown that BXG is abundant in bioactive substances that have a good regulatory effect on meat quality, including polysaccharides, flavonoids, and polyphenols. For example, polysaccharides from Camellia oleifera cake can improve the juiciness of broilers and change the meat color [8]. Seaweed polysaccharides can enhance the antioxidant capacity of pork [9], and dietary supplementation with Eucommia ulmoides Oliver polysaccharide can enhance the growth performance of Songliao Black Pigs and increase the levels of genes, thereby improving meat quality [10]. Dietary bamboo leaf flavonoids can enhance the antioxidant properties of meat, reduce fat oxidation, and improve the tenderness and color of chicken meat [11]. In addition, research has found that polyphenolic substances in pomegranate peels effectively increase the shelf life of meat products and reduce fat oxidation [12]. Polyphenolic substances in grape seeds can reduce fat oxidation and enhance the sensory quality of the meat [13]. Therefore, BXG, which is rich in bioactive substances, has great potential in improving meat quality.

Although the potential application value of BXG in animal feed has been preliminarily revealed, systematic research on its specific impact on the meat quality of finishing pigs is still insufficient. The polysaccharides, flavonoids, and polyphenols rich in BXG possess significant antioxidant, anti-inflammatory, and immunomodulatory properties, potentially enhancing the antioxidant capacity of pork and upregulating the expression of genes associated with meat quality, thus improving overall meat quality. Previous studies and preliminary experiments have demonstrated that substituting 10% of corn with BXG optimizes meat quality attributes without compromising animal health or performance. This substitution is both feasible and cost-effective for commercial applications [14]. Therefore, in this study, 10% of corn was replaced by BXG to make feed for finishing pigs, and the effects on carcass traits, meat quality, muscle amino acid profile, and gene expression levels of finishing pigs were systematically evaluated. Furthermore, proteomics technology was employed to investigate the various distinctions of proteins, providing practical guidance for utilizing BXG to produce higher-quality pork and promoting its commercial application.

## 2. Materials and Methods

### 2.1. Experimental Materials

BXG was supplied by Guangzhou Daqiao Food Equipment Co., Ltd. (Guangzhou, China). The flesh of the Passiflora edulis Sim was removed to obtain the peel, which was dried at 55 °C to maintain the bioactivity in BXG. Then, the dried BXG was crushed and sieved through a 200-mesh sieve to obtain powder [15]. The chemical composition analysis of BXG and the content of key amino acids are presented in Table 1 and Table S1.

### 2.2. Experimental Design and Diet

The Animal Care and Use Committee of the Guangdong Academy of Agricultural Sciences (authorization number GAASIAS-2022-072) approved all animal experiments.

In this experiment, 20 healthy Duroc × Landrace × Large finishing pigs (80.63 ± 0.255 kg) were randomly divided into two groups. Each group comprised 5 pens (replicates) with 2 pigs per pen. The control group (CON) was fed the basal diet, while the experimental group (BXG) received the basal diet with BXG instead of 10% corn for a period of 43 days. Throughout the entire feeding period, the finishing pigs were fed by specially assigned staff and fed freely, and the daily feed consumption was recorded. According to the nutritional requirements of finishing pigs, the full-nutrient diets of PE based on the balance of total nitrogen, energy, and key essential amino acids were designed and granulated. The diets of each group were configured based on the NRC (2012) nutritional requirements of pigs and the actual feeding situation (Table 2) [16].

### 2.3. Sample Collection

After 43 d of feeding, the pigs were fasted overnight (about 12 h), and one pig from each replication was randomly chosen (5 pigs/group) for slaughter, which was conducted using electrical stunning followed by exsanguination. The age and environmental conditions of all finishing pigs were standardized, and data were collected from five randomly selected pigs per group as detailed in Table S2 [17]. Post-slaughter, the *longissimus dorsi* muscle (LDM) was collected from the 3rd to 11th ribs on the left-side carcass, part of which was used for meat quality determination, and about 150 g of LDM was flash frozen in liquid nitrogen and stored at −80 °C for amino acid content, proteomics, and gene expression analysis.

### 2.4. Carcass Traits and Meat Quality

Post-slaughter, the carcass weight was recorded, and carcass yield was calculated. The weights of the heart, liver, and spleen were recorded, and organ indices were calculated. The backfat thickness of the first rib, waist, and last lumbar rib was measured with vernier calipers, and the average backfat thickness was calculated.

Meat quality was assessed as previously described [18]. pH value: A portable handheld pH meter (Hanna Instruments HI 9024C, Smithfield, RI, USA) was used to detect the pH value of the LDM (between the 10th and 11th ribs) at 45 min and 24 h post-slaughter. Each repetition was measured three times, and the average value was taken. Meat color: A portable handheld colorimeter (CR-400, Konica Minolta, Tokyo, Japan) was used to detect the meat color of the LDM at 45 min and 24 h post-slaughter. A CR-A44 calibration plate (serial number 16433029) was utilized. The illuminant was set to the D65 standard, and the observer angle was configured to the standard 2-degree setting. The equipment operates in a closed cone. Drip loss: A strip of LDM (approximately 55 g) from the 10th to 11th ribs was taken, and the weight was recorded 45 min post-slaughter. The sample was suspended using a fishhook within an inflated plastic bag, sealed, and stored at 4 °C for 24 h. After removing the sample, the moisture was blotted dry with filter paper, and the weight after 24 h was recorded to calculate the drip loss. Shear force: A strip of LDM (approximately 150 g) from the 10th to 11th ribs was taken 24 h post-slaughter and placed in a cooking bag, then boiled in a water bath until the center temperature reached 70 °C. After cooling to room temperature with running water, a strip of meat (1 cm × 1 cm × 3 cm) was taken along the direction of the muscle fibers. Finally, the shear force value of the meat piece was measured using a texture analyzer (C-LT3B, Brookfield, Shanghai, China). Intramuscular fat: The intramuscular fat content of the LDM from the 10th to 11th ribs was determined in accordance with the national standard method.

### 2.5. Antioxidant Status of Longissimus Dorsi

Accurately weigh the muscle sample (0.1 g), add 0.9 mL of physiological saline, place in a frozen grinder at 50 Hz for 2 min to prepare a 10% homogenate, then centrifuge at 4 °C, 2500 r/min for 10 min to obtain the supernatant. The content of catalase (CAT), malondialdehyde (MDA), total superoxide dismutase (T-SOD), and total antioxidant capacity (T-AOC) in the muscle was determined using a multifunctional microplate reader (Varioskan LUX, Singapore), according to the manufacturer’s instructions. The assay kits were purchased from Nanjing Jiancheng Bioengineering Institute (Nanjing, China).

### 2.6. Quantitative Real-Time PCR Analysis

The analysis was performed as previously described [19]. Total RNA from colonic mucosa was extracted using the TRIzol method. First, 100 mg of the sample was put in a 2.0 mL RNase-free EP tube, and 1 mL of Trizol (Takara Biotechnology, Dalian, China) was added. Subsequently, the mixture was put in a grinder to shake (60 Hz, 120 s), followed by resting on ice for 10 min. After discarding the supernatant, white RNA precipitate was visible at the bottom of the tube. Subsequently, 1 mL of 75% ethanol was added and then centrifuged at 4 °C, 12,000 r/min for 5 min, and the supernatant was discarded. After discarding the supernatant, the tube was dried in a clean bench. Depending on the amount of RNA precipitate, 20–50 μL of DEPC water was added to dissolve and mix. Finally, the concentration of RNA was determined using a microvolume spectrophotometer (NanoPhotometer^®^ N60, Implen, München, Germany).

RNA reverse transcription: Reverse transcription of RNA was performed using the EZBioscience^®^ (Roseville, MN, USA) Color Reverse Transcription Kit, synthesizing cDNA. The specific operation process was strictly carried out according to the instructions.

Primer design: The primer sequences for the target gene were devised using Primer 5.0 software and synthesized by Sangon Biotech (Shanghai) Co., Ltd. (Shanghai, China). The specific PCR primer sequences are presented in Table S3.

Real-time PCR: With cDNA as the template, following the reaction system in Table S4, the corresponding reagents were added in sequence into a 96-well quantitative PCR plate. Amplification was performed on the PCR instrument (Bio-Rad Laboratories, Hercules, CA, USA). The internal control gene β-actin was used for normalization. The mRNA expression levels of target genes were measured by the 2^−ΔΔCt^ method.

### 2.7. Determination of Free Amino Acid

Weigh the muscle sample (0.1 g), add 1 mL of 0.02 mol/L hydrochloric acid, mix well by vortexing, centrifuge (15,000 rpm, 4 °C, 15 min), take the supernatant and mix it with 10% sodium sulfosalicylate in a 1:1 ratio, centrifuge again (12,000 rpm, 4 °C, 20 min), filter through a 0.22 μm filter, and measure using an amino acid analyzer (L-8900, Hitachi Ltd., Tokyo, Japan).

### 2.8. TMT-Labelled Quantitative Proteomic Analysis

The LDM sample was fully ground into a fine powder under liquid nitrogen, then transferred to a 1.5 mL centrifugation tube; 600 μL of the extract solution was extracted with phenol added, and the final concentration of the extract was 1 mmol/L with PMSF, and the sample was broken by ultrasound on ice. Finally, the solution was centrifuged for 10 min to fully remove the precipitation. The protein concentration was determined by BCA protein concentration assay. Total protein of 100 μg was taken from each sample and added into DTT solution to make its final concentration of 4.5 mmol/L and incubated at 55 °C for 30 min. The precipitation was collected by centrifugation and dissolved with 100 μL of TEAB at 300 mmol/L, and 2 μg of Trypsin-TPGK was added and digested overnight at 37 °C. The freeze-dried samples were added with 66 μL of 200 mmol/L TEAB buffer, mixed well, and 30 μL samples were labeled with the TMT10plex^TM^ kit.

The labeled protein samples were separated by liquid chromatography (Agilent 1100 HPLC, Santa Clara, CA, USA) and subsequently collected in sequence for vacuum freeze drying prior to LC-MS/MS analysis. For separation, the samples were injected into an Acclaim PepMap RSLC column (RP-C18, Thermo Fisher, Waltham, MA, USA) at a flow rate of 300 nL/min. The eluents were mobile phase A (0.1% formic acid, formic acid/water = 0.1:99.9) and mobile phase B (80% acetonitrile, 0.1% formic acid, acetonitrile/water/formic acid = 80:19.9:0.1). Elution gradients were as follows: 0–4 min, 8–11% B; 4–36 min, 11–45% B; 36–39 min, 45–100% B; 39–45 min, 100% B. Mass spectrometry (MS) scanning was performed over a mass-to-charge ratio range of 350–1500 with a resolution of 60,000. The top 20 peaks in the MS spectrum were subjected to collision-induced dissociation with a normalized collision energy of 32. The MS/MS acquisition parameters included a resolution of 3000, an automatic gain control target of 200,000, a maximum ion injection time of 40 ms, and a dynamic exclusion duration of 30 s.

### 2.9. Statistics and Analysis of Data

Excel software was used to classify and organize the experimental data. After checking for normal distribution by the Shapiro–Wilk test, the SPSS 23.0 software (IBM Corporation, Armonk, NY, USA) was used to perform an independent sample t-test, and a graphical representation was created using GraphPad Prism 8.0.2. The results were denoted as the mean ± standard error of the mean (SEM), with *p* < 0.05 indicating that the difference was significant, and 0.05 ≤ *p* < 0.10 indicating that there was a tendency for change.

Proteomic raw data were pre-processed using ProteomeDiscoverer (v.2.4.1). Principal component analysis (PCA) was performed using R package ropls (v.1.6.2). Proteins with a *p*-value < 0.05 based on an unpaired Student’s t-test and a fold change (FC) value ≥ 1.2 or ≤0.83 were identified as differentially abundant proteins. The Kyoto encyclopedia (KEGG) database was used for enrichment analysis.

## 3. Results

### 3.1. Carcass Traits

As presented in Table 3, compared to the CON group, the carcass weight of the BXG group was significantly increased (*p* < 0.05), while there were no significant differences in carcass yield, average backfat thickness, leaf lard, heart, liver, and spleen weights (*p* > 0.05).

### 3.2. Meat Quality

As presented in Table 4, the BXG group exhibited lower drip loss, shear force, b*_45min,_ and b*_24h_ values compared to the CON group (*p* < 0.05), while marbling scores and intramuscular fat content were significantly increased (*p* < 0.05). There were no significant differences in other meat quality indicators between the two groups (*p* > 0.05).

### 3.3. Antioxidant Indices

As presented in Table 5. The BXG group had no significant effect on the antioxidant indicators of finishing pigs compared to the CON group (*p* > 0.05).

### 3.4. Free Amino Acid Profile

The BXG group had a significant decrease in the content of glutamine, glutamate, proline, methionine, tyrosine, and isoleucine compared to the CON group (*p* < 0.05), while there was no significant difference in the content of other amino acids (*p* > 0.05) (Table 6).

### 3.5. mRNA Expressions of Genes

**Figure 1:**
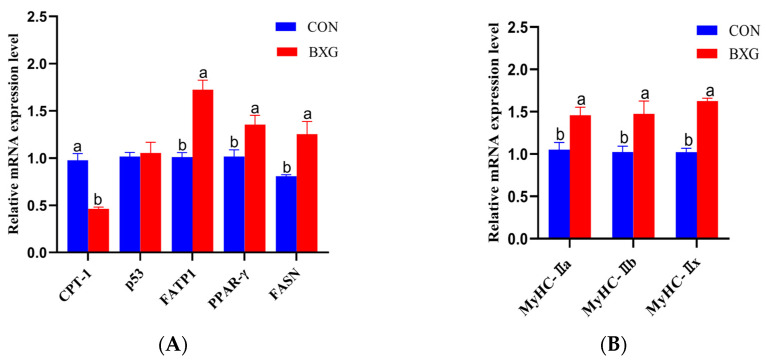
The mRNA expression of genes in the LDM of finishing pigs (**A**,**B**). FASN, fatty acid synthase; PPARγ, peroxisome proliferator-activated receptor γ; FATP1, fatty acid transport protein 1; CPT-1, carnitine palmitoyl transferase 1; MyHC-I, myosin heavy chain I; MyHC-IIa, myosin heavy chain IIa; MyHC-IIb, myosin heavy chain IIb; MyHC-IIx, myosin heavy chain IIx. Data are mean and SEM (n = 5). ^a,b^ mean values with different superscripts differ in the same row (*p* < 0.05).

As presented in Figure 1A, the mRNA levels of FATP1, PPAR-γ, and FASN were significantly increased in the BXG group, while CPT-1 mRNA expression was decreased (*p* < 0.05). The level of p53 mRNA was not different in the CON and BXG groups (*p* > 0.05).

As shown in Figure 1B, the mRNA levels of MyHC-1, MyHC-IIa, and MyHC-IIx mRNA were significantly increased, while the expression level of MyHC-IIb mRNA was significantly decreased in the BXG group (*p* < 0.05).

### 3.6. Proteomic Analysis

**Figure 2:**
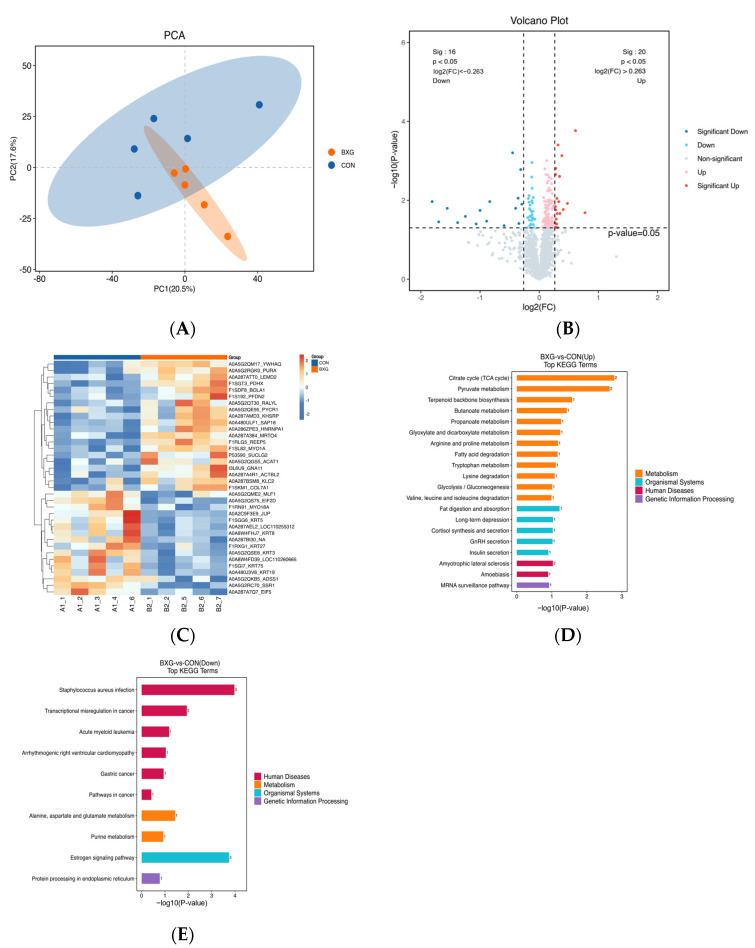
The proteomic profiles of LDM (n = 5). (**A**) Principal component analysis (PCA) plot. (**B**) Differential protein volcano plots. (**C**) Hierarchical clustering of differential proteins. Red indicates highly expressed proteins, blue indicates low-expressed proteins, and each line represents the relative expression levels of the proteins across different groups. (**D**,**E**) KEGG pathway enrichment analysis of differential proteins.

As presented in Figure 2A, the PCA model showed that PC1 and PC2 were 20.5% and 17.6%, respectively. The CON and BXG groups were spatially separated, suggesting that the addition of BXG resulted in significant metabolic changes.

A total of 36 differentially expressed proteins were identified between the CON and BXG groups, with 20 proteins showing increased expression, while 16 proteins exhibited decreased expression in the BXG group. The top five upregulated proteins included receptor accessory protein 5 (REEP5), Myosin1A (MYO1A), Guanine Nucleotide-Binding Protein Subunit alpha-11 (GNA11), kinesin light chain 2 (KLC2), and succinate-CoA ligase GDP-forming beta subunit (SUCLG2). As shown in Figure 2B, the quantitative data of the two groups were presented in the form of a volcano plot. According to the significance of the different proteins, the details of the intermediate and downregulated proteins in the two groups are shown in Table S5.

The clustering of differentially expressed proteins is shown in Figure 2C. The changes in different proteins between groups were directly demonstrated, and it could be seen that the expression of some proteins in the two groups was significantly different.

Differential protein enrichment pathways were analyzed, as shown in Figure 2D,E, revealing that the significantly upregulated pathways mainly involve metabolic pathways (such as pyruvate metabolism, the tricarboxylic acid cycle, fatty acid degradation, and glycolysis) and endocrine pathways (such as insulin secretion and GnRH secretion). The significantly downregulated pathways mainly include human disease-related pathways (such as cancer-related pathways, acute myeloid leukemia) and metabolic pathways (such as aspartate and glutamate metabolism, purine metabolism).

## 4. Discussion

### 4.1. Carcass Traits and Meat Quality of Finishing Pigs Were Altered by BXG

In this study, 10% of corn was replaced by BXG and showed a positive effect on improving the carcass weight. Although the carcass weight was significantly increased in the BXG group, there were no significant differences between the two groups in backfat thickness, leaf lard, and the weight of the heart, liver, and spleen. The results indicated that the basic structure and visceral weight of the carcass had no significant differences with the BXG addition, which is consistent with partial findings that suggest that the impact of plant-based additives on carcass traits may be relatively mild [20]. Togashi et al. found that the carcass weight, backfat thickness, leaf lard, and the weight of the heart, liver, and spleen had no significant differences by adding 4% and 8% passion fruit seed powder to broiler diets, which is consistent with our previous study [21]. However, although there were no significant changes in carcass yield, further research is warranted into the improvement of meat quality and fat distribution.

Meat quality is an important economic indicator that influences consumers’ willingness to purchase livestock production. At present, the evaluation indexes of pork mainly include pH value, IMF content, drip loss, shear force, and meat color. In this study, the marbling scores and IMF contents of LDMs were significantly increased, while b*_45min_, b*_24h_, drip loss_24h,_ and shear force were significantly reduced in the BXG group. Excessive drip loss can lower the meat color a* value and increase the b* value, leading to the production of pale soft exudative (PSE) meat. The IMF content and shear force are negatively correlated, and higher IMF content results in lower shear force values, indicating more tender meat [22]. The shear force results of the BXG group indicate that the meat was more tender and smooth, which may be attributed to the natural compounds (such as antioxidants and phenolic substances) richly contained in the BXG. These components can promote the softening of muscle fibers, thereby improving the tenderness of the meat [17]. As a natural additive of plant origin, the improvement in the meat quality of the BXG group may be closely related to its rich polyphenols, dietary fiber, and antioxidant components. Polyphenols are known to regulate fat metabolism, enhance meat water-holding capacity, and increase antioxidant capacity [23]. Therefore, this study showed that adding BXG to feed can partially improve the quality characteristics of fattening pork, especially in reducing drip loss and shear force, while also enhancing marbling and increasing intramuscular fat content, showing high application potential.

The amino acid results indicated that the levels of glutamine, glutamate, proline, and others were reduced in the BXG group, which may be related to the use of 10% of corn being replaced by BXG to feed for finishing pigs in this study. As a common feed source, corn contains a variety of amino acids [24]. In the present study, five essential amino acids (lysine, methionine, threonine, tryptophan, and valine) were balanced in the diets of the two groups, but the content of other amino acids was not balanced, which may also lead to the decrease in the above amino acid content in pig muscle. Secondly, the small sample size or the limited number of groups will also have a certain impact on the result. However, the decrease in total free amino acids in the BXG group may imply an increased utilization of amino acids in other metabolic pathways. For example, the involvement of amino acids in the TCA cycle may increase energy supply, thereby supporting higher muscle metabolic activity [25]. Therefore, although the content of some amino acids was reduced, the dietary fiber, antioxidant components, and phytochemical components in BXG may affect the absorption and metabolism of amino acids through complex biological mechanisms, and the improvement of the overall metabolism level was helpful to maintain or even improve the texture of meat. Accordingly, it is suggested that in future studies, these amino acids should be supplemented, or the proportion of BXG in the diet should be decreased to eliminate the negative effects.

### 4.2. The mRNA Expression of Lipid Metabolism and Muscle Fiber Types in the LDM of Finishing Pigs Was Altered by BXG

The IMF content is closely related to meat quality, especially in terms of juiciness and tenderness [26]. In this study, the IMF content was increased in the BXG group, indicating a beneficial effect on meat quality. Additionally, genes related to lipid metabolism (FATP1, PPAR-γ, FASN, and CPT-1) were regulated with BXG treatment. FATP1, PPAR-γ, and FASN are involved in the transport, storage, and synthesis of fatty acids, while CPT-1 is involved in the transport and oxidation of fatty acids [27]. In the BXG group, the mRNA expression of FATP1, PPAR-γ, and FASN was significantly increased, suggesting that BXG may accelerate IMF deposition by promoting fatty acid transport, adipocyte differentiation, and fatty acid synthesis pathways [28,29]. However, the decreased mRNA expression of CPT-1, a key enzyme for fatty acids to enter the mitochondria for oxidation, may indicate that BXG inhibits fatty acid oxidation, thereby promoting fat accumulation [30]. Studies have shown that plant extracts have potential application value in improving meat quality, particularly in regulating fat deposition [31].

The makeup and proportion of different types of muscle fibers significantly influence meat quality parameters such as color, pH value, and tenderness [32]. Based on the distinct subtypes of myosin heavy chain, the muscle fibers are categorized into four types, namely type I (slow oxidizing type, MyHC-I), type IIa (fast oxidizing type, MyHC-IIa), type IIb (fast enzymatic type, MyHC-IIb), and type IIx (intermediate type, MyHC-IIx). Oxidized muscle fibers (type I, type IIa) have smaller diameters and higher myoglobin content, so when the proportion of oxidized muscle fibers is higher, the muscle color is bright red, and the flesh color score is higher [33]. At the same time, some studies found that the diameter of muscle fibers is related to meat quality. Specifically, finer and denser muscle fibers are associated with higher intramuscular fat content, resulting in more tender meat. However, an increase in the proportion of glycolytic fibers (type IIb) will increase the shear force within the muscle, thereby reducing the tenderness of the meat [34]. The results showed that the mRNA levels of MyHC-I and MyHC-IIa were significantly elevated in the BXG group, while the mRNA expression of MyHC-IIb was markedly reduced, indicating a greater proportion of oxidized muscle fibers. Correspondingly, the BXG group exhibited a higher IFM, along with lower b* values and shear force in the LDM. Therefore, this study indicated that BXG can not only promote fat deposition but also may further optimize meat quality characteristics by changing muscle fiber composition.

### 4.3. The Protein Expression in the LDM of Finishing Pig Was Altered by BXG

A quantitative proteomic analysis based on TMT was used to compare the proteins that were differently expressed in the two groups. This study showed that the top five upregulated proteins included REEP5, MYO1A, GNA11, KLC2, and SUCLG2. MYO1A is an ATP-independent myosin that is involved in cell movement and substance transport, especially in the muscle contraction and structural maintenance of muscle fibers. The enhanced expression of MYO1A may improve the stability of muscle fibers and the tenderness and taste of meat [35]. This was in line with the BXG muscles having a larger percentage of oxidized muscle fibers. KLC2 is a light chain protein in the kinesin family, and the upregulation of KLC2 may increase the anabolic activity of muscles, resulting in increased muscle weight and density, contributing to improved carcass performance [36]. This was consistent with the increase in the carcass weight of fattened pigs in the BXG group. GNA11, a G protein subunit, is involved in signal transduction [37]. SUCLG2 is one of the subunits of succinate-CoA synthetase, which significantly influences ATP production and fatty acid oxidation [38]. In general, MYO1A and KLC2 upregulation contribute to improved muscle tenderness and structural stability, which enhances meat’s ability to hold its shape during processing. The upregulation of GNA11 and SUCLG2 may improve the nutritional value and flavor of meat by enhancing fat metabolism and energy supply. KEGG pathway analysis indicated that there was a notable increase in the activity of the TCA cycle and pyruvate metabolic processes in the BXG group. The TCA cycle and pyruvate metabolism are the core pathways of cell energy generation, and the enhancement of the TCA cycle activity helps to increase the production of ATP and improve the metabolic efficiency of cells. Its upregulation usually implies a higher level of energy metabolism [39]. Enhanced energy metabolism may improve the nutritional value of meat and increase metabolite storage in muscle cells, thereby contributing to enhanced meat quality characteristics such as tenderness and juiciness, resulting in a more intense flavor [40]. In addition, the increased activity of the TCA cycle may reduce lactic acid accumulation in the muscle and improve the commercial value of the carcass. In conclusion, dietary BXG supplementation can improve meat quality by changing metabolic pathways, especially in energy metabolism. These changes may contribute to enhancing the nutritional composition and flavor characteristics of meat, providing potential applications for meat processing and production. Future studies should further investigate the specific active components in passion fruit peel and their metabolic regulatory mechanisms in order to more fully understand their effects on meat quality.

## 5. Conclusions

In summary, although partly replacing the corn with BXG in the feed composition could significantly improve the meat quality, the content of some amino acids in muscle was decreased. Furthermore, dietary BXG supplementation could help to upregulate the mRNA expression of genes related to lipid metabolism and oxidized muscle fibers and improve meat quality by altering metabolic types such as energy metabolism. These results indicate that passion fruit peel has the potential to be used as a plant-derived additive to improve pork quality. However, it is necessary to balance some key amino acids or optimize the additional amount and ratio of this kind of fruit residue to maintain pork flavor.

## Figures and Tables

**Table 1:** Chemical composition analysis of BXG.

Items	Value
Crude fiber	26.3%
Neutral detergent fiber	35.2%
Total phosphorus	0.24%
Moisture content	6.6%
Crude ash	9.0%
Crude fat	0.9%
Crude protein	10.06%
Calcium	0.394%
GE	3429 kcal/kg

**Table 2:** Feed ingredient and nutrient composition of experimental diets (as-fed basis).

Items	Dietary Treatment	
	CON	BXG
Ingredients	as-fed basis, %	
Corn	71.35	62.55
Soybean meal	19.92	20.23
Soybean oil	0.25	2.75
Passiflora edulis pomace	0.00	10.00
Beancurd sheet	6.00	2.00
Calcium carbonate	0.73	0.71
Dicalcium phosphate	0.45	0.46
Sodium chloride	0.18	0.18
Choline chloride	0.10	0.10
Phytase	0.02	0.02
Vitamin–mineral premix ^1^	1.00	1.00
Total	100.00	100.00
Nutrient levels ^2^		
GE, kcal/kg	3326	4010
NE, kcal/kg	2400	2400
Crude protein	16.00	16.00
Calcium	0.47	0.47
Digestible phosphorus	0.22	0.22
Digestible lysine	0.680	0.675
Digestible methionine	0.231	0.231
Digestible threonine	0.483	0.481
Digestible tryptophan	0.157	0.157
Digestible valine	0.627	0.625

^1^ Vitamin–mineral premix provided per kilogram of complete diet: vitamin A, 6500 IU; vitamin D3, 2000 IU; vitamin E, 150 mg; vitamin K3, 3 mg; vitamin B12, 0.03 mg; vitamin B1, 3 mg; vitamin B2, 6 mg; vitamin B6, 5 mg; nicotinic acid, 45 mg; D-pantothenic acid, 9 mg; folic acid, 1 mg; biotin, 0.3 mg; Fe (FeSO_4_·H_2_O), 72 mg; Cu (CuSO_4_·5H_2_O), 10 mg; Mn (MnSO_4_·H_2_O), 42 mg; Zn (ZnSO_4_·H_2_O), 72 mg; I (KI), 0.42 mg; Se (Na_2_SeO_3_), 0.2 mg; Mg (MgO), 34 mg. ^2^ The measurements for crude protein and crude fiber were obtained directly, whereas the remaining values were derived through calculation.

**Table 3:** Effect of BXG on carcass traits of finishing pigs.

Items	CON	BXG	*p*-Value
Carcass weight/kg	91.25 ± 0.90 ^b^	94.73 ± 0.44 ^a^	0.017
Carcass yield/%	77.79 ± 0.55	76.02 ± 0.56	0.102
Average backfat thickness/cm	3.27 ± 0.06	3.13 ± 0.16	0.230
Leaf lard/kg	2.60 ± 0.15	1.93 ± 0.31	0.062
Heart weight/kg	0.51 ± 0.03	0.50 ± 0.02	0.836
Liver weight/kg	1.95 ± 0.04	1.94 ± 0.05	0.896
Spleen weight/kg	0.25 ± 0.03	0.25 ± 0.02	0.811

Data are mean and SEM (n = 5). ^a,b^ mean values with different superscripts differ in the same row (*p* < 0.05).

**Table 4:** Effect of BXG on meat quality of finishing pigs.

Items	CON	BXG	*p*-Value
pH_45min_	6.26 ± 0.07	6.13 ± 0.12	0.446
pH_24h_	5.45 ± 0.11	5.46 ± 0.06	0.886
pH_48h_	5.42 ± 0.04	5.45 ± 0.05	0.559
L* (lightness)_45min_	45.94 ± 0.75	44.92 ± 0.96	0.477
a* (redness)_45min_	17.05 ± 0.32	17.21 ± 0.53	0.824
b* (yellowness)_45min_	3.22 ± 0.36 ^a^	2.18 ± 0.17 ^b^	0.048
L* (lightness)_24h_	55.76 ± 1.14	54.67 ± 0.94	0.528
a* (redness)_24h_	16.35 ± 0.47	15.85 ± 0.45	0.515
b* (yellowness) _4h_	3.82 ± 0.19 ^a^	3.04 ± 0.18 ^b^	0.029
L* (lightness)_48h_	55.70 ± 1.46	55.68 ± 1.07	0.990
a* (redness)_48h_	16.83 ± 0.57	16.90 ± 0.55	0.942
b* (yellowness)_48h_	3.42 ± 0.36	3.19 ± 0.21	0.639
Drip loss_24h_, %	3.17 ± 0.10 ^a^	2.83 ± 0.08 ^b^	0.046
Drip loss_48h_, %	3.66 ± 0.15	3.43 ± 0.13	0.321
Shear force, N	76.81 ± 3.50 ^a^	64.22 ± 2.63 ^b^	0.033
Marbling scores	2.52 ± 0.16 ^b^	3.10 ± 0.12 ^a^	0.044
IMF content, %	1.50 ± 0.23 ^b^	2.58 ± 0.36 ^a^	0.048

Data are mean and SEM (n = 5). ^a,b^ mean values with different superscripts differ in the same row (*p* < 0.05).

**Table 5:** Effect of BXG on antioxidant status of longissimus dorsi in finishing pigs.

Items	CON	BXG	*p*-Value
T-AOC, nmol/g prot	0.09 ± 0.01	0.09 ± 0.01	0.594
CAT, U/mgprot	1.45 ± 0.16	1.24 ± 0.13	0.375
MDA, nmol/mgprot	0.27 ± 0.03	0.30 ± 0.05	0.737
SOD, U/mgprot	89.09 ± 3.50	81.51 ± 3.67	0.230

Data are mean and SEM (n = 5). Abbreviations: CAT, catalase; T-AOC, total antioxidant capacity; MDA, malondialdehyde; SOD, superoxide dismutase.

**Table 6:** Effect of BXG on free amino acids of the LDM.

Items	CON	BXG	*p*-Value
Histidine	1.53 ± 0.02	1.56 ± 0.15	0.832
Arginine	7.50 ± 0.12	7.72 ± 0.29	0.531
Asparagine	7.37 ± 0.08	7.37 ± 0.56	0.995
Glutamine	580.99 ± 19.27 ^a^	494.50 ± 7.57 ^b^	0.006
Serine	8.33 ± 0.07	9.43 ± 0.53	0.105
Glycine	46.72 ± 0.77	48.39 ± 0.76	0.206
Aspartic acid	5.32 ± 0.34	5.40 ± 0.27	0.880
Glutamic acid	12.27 ± 0.28 ^a^	8.31 ± 0.13 ^b^	<0.001
Threonine	7.44 ± 0.04	7.18 ± 0.35	0.529
Alanine	55.27 ± 0.49	60.61 ± 3.59	0.224
Proline	12.52 ± 0.11 ^a^	11.92 ± 0.07 ^b^	0.004
Lysine	8.22 ± 0.16	8.76 ± 0.73	0.536
Methionine	6.33 ± 0.26 ^a^	4.75 ± 0.30 ^b^	0.008
Tyrosine	10.54 ± 0.06 ^a^	8.13 ± 0.54 ^b^	0.004
Valine	10.48 ± 0.26	11.13 ± 1.22	0.653
Isoleucine	6.94 ± 0.23 ^a^	6.10 ± 0.11 ^b^	0.018
Leucine	13.00 ± 0.14	12.28 ± 1.41	0.658
Phenylalanine	10.34 ± 0.21	9.30 ± 0.76	0.272
Tryptophan	2.74 ± 0.15	2.69 ± 0.14	0.851
Total FAAS	816.17 ± 22.68 ^a^	727.30 ± 18.21 ^b^	0.026
FAA ^1^	140.47 ± 2.08	140.14 ± 5.74	0.936
EAA ^2^	67.02 ± 1.39	63.75 ± 4.93	0.584

Data are mean and SEM (n = 5). ^a,b^ Mean values with different superscripts differ in the same row (*p* < 0.05). Abbreviations: FAAs, free amino acids; EAAs, essential amino acids; FAAs, flavor amino acids. ^1^ Flavor amino acids = glycine + aspartic acid + alanine + glutamic acid + tyrosine; ^2^ essential amino acids = histidine + threonine + lysine + methionine + valine + isoleucine + leucine + phenylalanine + tryptophan.

## Data Availability

The original contributions presented in the study are included in the article/Supplementary Material, further inquiries can be directed to the corresponding author.

## Supplementary Materials

The following supporting information can be downloaded at: https://www.mdpi.com/article/10.3390/foods14040561/s1, Table S1: Hydrolyzed amino acid content of BXG; Table S2: Weight data for a group of 10 pigs and a subgroup of 5 selected pigs; Table S3: Primers used in this study; Table S4: Real-time quantitative PCR reaction system; Table S5: The differential protein between the CON and BXG groups.
