# Supplementary material for: Effect of Passiflora Edulis Sims Peel Feed on Meat Quality of Finishing Pigs
Source: Foods. 2025 Feb 8;14(4):561. doi: 10.3390/foods14040561 (PMC11854249; doi:10.3390/foods14040561)
Supplement: Supplementary file 1 [file foods-14-00561-s001.zip › foods-3403256-supplementary.pdf]

Table legends:

Supplemental Table S1 Hydrolyzed amino acid content of BXG.

Supplemental Table S2 Weight data for a group of 10 pigs and a subgroup of 5 selected pigs.

Supplemental Table S3 Primers used in this study.

Supplemental Table S4 Real-time quantitative PCR reaction system.

Supplemental Table S5 The differential protein between the CON and BXG groups.

Supplemental Table S1

Hydrolyzed amino acid content of BXG

Items	Content%
Threonine	0.227
Methionine	0.01
Valine	0.231
Phenylalanine	0.224
Leucine	0.317
Lysine	0.345
Aspartic acid	1.035
Serine	0.302
Glutamic acid	2.421
Glycine	0.278
Alanine	0.344
Cysteine	0.01
Isoleucine	0.167
Tyrosine	0.096
Histidine	0.118
Arginine	0.253
Proline	0.377

Supplemental Table S2

Weight data for a group of 10 pigs and a subgroup of 5 selected pigs

Number	Final weight/kg	Number	Final weight/kg
CON-1	119.4	BXG-1	124.8
CON-2	119.6	BXG-2	122.0
CON-3	118.5	BXG-3	124.2
CON-4	114.8	BXG-4	126.8
CON-5	117.7	BXG-5	126.7
CON-6	119.5	BXG-6	125.0
CON-7	115.1	BXG-7	122.4
CON-8	114.8	BXG-8	122.5
CON-9	115.0	BXG-9	126.8
CON-10	115.8	BXG-10	127.0
mean ± SEM	117.02 ± 0.63	mean ± SEM	124.82 ± 0.60

Number	Final weight/kg	Number	Final weight/kg
CON-1	119.4	BXG-1	124.8
CON-3	118.5	BXG-3	124.2
CON-5	117.7	BXG-5	126.7
CON-7	115.1	BXG-6	125.0
CON-10	115.8	BXG-8	122.5
mean ± SEM	117.30 ± 0.72	mean ± SEM	124.64 ± 0.61

Supplemental Table S3

Primers used in this study.

Gene	Nucleotide sequence (5-3′)
FASN	Forward: TGGAGGTGCGCCAGATAC<br>Reverse: TCCTTGGAACCGTCTGTGTTC
p53	Forward: GGTTCAGCGTTGCCTGCT<br>Reverse: CGCAAGACGGCGGATTTA
PPAR-γ	Forward: GATTTCTCCAGCATTTCCA<br>Reverse: GCTCTTCGTGAGGTTTGTT
CPT-1	Forward: CTGAGAGATATGGCCCCTACTG<br>Reverse: CCAGGTCCATGACGTAATAGTTG
FATP-1	Forward: TCTGCGTCGCTTTGATGGC<br>Reverse: GCCGTGAGGGTCTGCGAT
MyHC-IIa	Forward: AAACCTCACGGAAGAGATGG<br>Reverse: TCAGGGTGTTGACTTTGTCCT
MyHC-IIx	Forward: GAAACCGTCAAGGGTCTACG<br>Reverse: CGCTTCCTCAGCTTGTCTCT
MyHC-IIb	Forward: GATGTTCCTGTGGATGGTCA<br>Reverse: CTCGTTGGTGAAGTTGATGC
β-actin	Forward: GGACTTCGAGCAGGAGATGG<br>Reverse: GCACCGTGTTGGCGTAGAGG

Supplemental Table S4

Real-time quantitative PCR reaction system

Reagent	Dose（μl）
PCR Forward Primer	0.4
PCR Reverse Primer	0.4
TB GreenTM Premix Ex TaqTM	10.0
cDNA	2.0
ddH2O	7.2

Supplemental Table S5

The differential protein between the CON and BXG groups.

Accession	Gene Name	BXG	CON	P-value	FC(BXG/CON)	Regulate
A0A5G2RC70	SSR1	15.107228	15.557052	0.00062579	0.732132158	Down
A0A5G2QS75	EIF2D	13.696766	14.009614	0.001666269	0.805050952	Down
A0A287A7Q7	EIF5	16.634512	16.99154	0.008852592	0.780771339	Down
A0A5G2QSE8	KRT3	17.479696	18.315604	0.010778203	0.560230332	Down
F1SGI7	KRT75	15.953814	17.762494	0.010778353	0.285451985	Down
A0A287BI30	NA	16.29664	16.591048	0.012557023	0.81540686	Down
A0A480J3V8	KRT19	13.432344	14.986936	0.015960593	0.340424789	Down
F1RN91	MYO18A	13.345786	13.744058	0.015977243	0.758766558	Down
A0A2C9F3E9	JUP	16.282032	17.281782	0.018034606	0.500086651	Down
A0A287AEL2	LOC110255312	19.046888	20.294912	0.025616547	0.421024473	Down
F1RXG1	KRT27	15.052642	15.940992	0.033794656	0.540231624	Down
A0A8W4FD39	LOC110260665	14.636112	16.334722	0.035188944	0.30808279	Down
F1SGG6	KRT5	18.68056	20.059148	0.036782293	0.384595023	Down
A0A5G2QME2	MLF1	13.173156	13.516872	0.038755027	0.788008993	Down
A0A8W4FHJ7	KRT8	15.292552	16.358736	0.039737891	0.477580554	Down
A0A5G2QKB5	ADSS1	16.44445	17.037044	0.043881968	0.663149476	Down
F1RLG5	REEP5	17.620694	17.007744	0.000172052	1.529383273	Up
F1SGT3	PDHX	18.492542	18.17432	0.000395598	1.246793034	Up
F1SL82	MYO1A	15.776254	15.393736	0.000740087	1.303615129	Up
A0A480ULF1	SAP18	15.302088	15.024304	0.001552971	1.212331295	Up
A0A287A384	MRTO4	11.699148	11.424502	0.002197233	1.209697224	Up
A0A5G2QM17	YWHAQ	15.596166	15.252964	0.002481604	1.268569007	Up
A0A5G2RGK0	PURA	17.157468	16.859334	0.008969262	1.229553065	Up
A0A286ZPE3	HNRNPA1	18.704438	18.374568	0.010839963	1.256900111	Up
I3L6U9	GNA11	15.85681	15.38023	0.012010048	1.391441254	Up
A0A287A4R1	ACTBL2	13.794328	13.521844	0.014455438	1.207885749	Up

F1SDF8	BOLA1	15.834808	15.429412	0.017143284	1.324452405	Up
A0A287ATT0	LEMD2	14.604734	14.32614	0.017526422	1.213012149	Up
A0A287BSM8	KLC2	16.85319	16.080042	0.020787368	1.708994791	Up
P53590	SUCLG2	17.332202	16.981506	0.021645019	1.275175663	Up
A0A287AMD3	KHSRP	15.063018	14.760296	0.021696912	1.233469464	Up
A0A5G2QGS5	ACAT1	19.412514	19.1252	0.029982403	1.220366092	Up
A0A5G2QT30	RALYL	16.35507	16.085654	0.038182952	1.205319818	Up
F1SKM1	COL7A1	14.405678	14.111574	0.039803988	1.226123245	Up
A0A5G2QE95	PYCR1	13.607822	13.33436	0.04826185	1.20870485	Up
F1S192	PFDN2	16.914456	16.60818	0.049129947	1.236511794	Up
